# Pitfalls in Scalp High-Frequency Oscillation Detection From Long-Term EEG Monitoring

**DOI:** 10.3389/fneur.2020.00432

**Published:** 2020-06-02

**Authors:** Nathalie Gerner, Aljoscha Thomschewski, Adrian Marcu, Eugen Trinka, Yvonne Höller

**Affiliations:** ^1^Department of Neurology, Christian-Doppler Medical Centre, Centre for Cognitive Neuroscience, Paracelsus Medical University, Salzburg, Austria; ^2^Department of Mathematics, Paris-Lodron University of Salzburg, Salzburg, Austria; ^3^Department of Psychology, University of Akureyri, Akureyri, Iceland

**Keywords:** high-frequency oscillations, HFO detection, electroencephalography, video-EEG monitoring, long-term EEG, epilepsy

## Abstract

**Aims:** Intracranially recorded high-frequency oscillations (>80 Hz) are considered a candidate epilepsy biomarker. Recent studies claimed their detectability on the scalp surface. We aimed to investigate the applicability of high-frequency oscillation analysis to routine surface EEG obtained at an epilepsy monitoring unit.

**Methods:** We retrospectively analyzed surface EEGs of 18 patients with focal epilepsy and six controls, recorded during sleep under maximal medication withdrawal. As a proof of principle, the occurrence of motor task-related events during wakefulness was analyzed in a subsample of six patients with seizure- or syncope-related motor symptoms. Ripples (80–250 Hz) and fast ripples (>250 Hz) were identified by semi-automatic detection. Using semi-parametric statistics, differences in spontaneous and task-related occurrence rates were examined within subjects and between diagnostic groups considering the factors diagnosis, brain region, ripple type, and task condition.

**Results:** We detected high-frequency oscillations in 17 out of 18 patients and in four out of six controls. Results did not show statistically significant differences in the mean rates of event occurrences, neither regarding the laterality of the epileptic focus, nor with respect to active and inactive task conditions, or the moving hand laterality. Significant differences in general spontaneous incidence [WTS(1) = 9.594; *p* = 0.005] that indicated higher rates of fast ripples compared to ripples, notably in patients with epilepsy compared to the control group, may be explained by variations in data quality.

**Conclusion:** The current analysis methods are prone to biases. A common agreement on a standard operating procedure is needed to ensure reliable and economic detection of high-frequency oscillations.

## Introduction

High-frequency oscillations (HFOs) in the EEG are fast local oscillatory field potentials, commonly sub-classified as ripples (80–250 Hz) and fast ripples (>250 Hz) (1). Intracranially recorded HFOs are considered a candidate epilepsy biomarker (2). More recently, HFOs have been claimed to be also detectable on the surface and scalp HFO analysis has been discussed as a potential tool for epilepsy screening (3, 4). In order to pave the way for clinical application in the routine environment of an epilepsy monitoring unit, the differentiation of pathological HFOs not only from artifacts but also from physiological HFOs is considered a key issue (5).

Scalp HFOs were predominantly detected interictally during non-rapid eye movement sleep in patients with focal epilepsy associated with traditional markers and the seizure-generating zone (3, 6). However, findings appear ambiguous, as HFOs, primarily suggested to be a specific marker of the epileptic focus, did not differentiate between focal and generalized epilepsy (7). The relationship of HFOs with the epileptic focus and traditional markers must not be exclusive. For example, intracranial HFOs preceding ictal spasm have been shown to not exclusively map the seizure-onset zone but also reflect the occurrence of motor symptoms in neighboring areas (8); similarly, scalp HFOs were more closely correlated with seizure frequency than traditional markers (9). Besides this, the differentiation of pathological and physiological HFOs within subject is largely unexplored in scalp EEG.

Above all, non-cerebral noise largely overlaps with the signal of interest (10, 11); it is argued that a successful detection of these small-scale events on the scalp surface is primarily dependent on a high signal-to-noise ratio (12). Studies comparing simultaneous depth-electrode and subdural recordings (13) as well as subdural and scalp recordings (4) reported reduced sensitivity from brain to scalp—not least due to spatial undersampling. It is debatable to which extent clinical routine EEGs can provide sufficient data quality.

Physiological HFOs, systematically elicited, could provide a proof of principle. Simple motor tasks constitute favorable prerequisites for scalp HFO detection, ensuring minimal signal-to-sensor distance over brain regions with minimal muscle activity. A first approach using a motor task to distinguish physiological from pathological HFOs in intracranial EEGs of patients with an epileptic focus outside the motor cortex led to ambiguous results (14). Research on evoked HFOs, where noise is canceled out by averaging across trials, however, provides essential information: High-frequency activity in central regions was associated with self-paced movement; as shown in intracranial and surface EEG (15–17) by common activation patterns contralateral to the moving body part in patients and healthy subjects.

Against this background, the question arises whether spontaneously occurring HFOs can be detected in scalp EEGs recorded within the clinical routine, and whether they can be used for epilepsy screening. To this end, (I) HFOs must be distinguishable from noise; (II) HFOs must be distinguishable between subjects with and without epilepsy; and (III) pathological HFOs must be distinguishable from physiological HFOs within subjects. We assume that (I) and (II) are achieved when the spontaneous incidence of HFOs differs systematically between diagnostic groups and across brain regions, topographically consistent with the diagnosed epileptic focus. In contrast, we assume that (III) can be achieved if physiological HFOs are evoked by task performance with a corresponding response pattern. Thus, we hypothesize that we can identify (i) spontaneously occurring HFOs over brain areas that correspond with the epileptic focus in patients with epilepsy and (ii) HFOs induced by self-paced motor activity in movement-associated central brain areas, independently of diagnosis.

## Methods

### Participants

We retrospectively analyzed EEG recordings from patients admitted to the epilepsy monitoring unit of the Department of Neurology, Christian-Doppler Medical Centre Salzburg. The described retrospective analysis was conducted on data collected as part of a clinical trial on the effects of epileptic seizures on memory performance. The respective study was conducted in accordance with the recommendations for Good Clinical Practice and was approved by the local ethics committee (Ethik-Kommission für das Bundesland Salzburg: E/1755, initial approval on 30/03/2014, latest amendment on 11/07/2016). Written informed consent was given by each patient prior to the experimental session.

Between November 2015 and May 2017, 63 patients had been recruited to take part in the aforementioned study. For the purpose of this scalp HFO analysis, we excluded two patients with intracranial recordings, 19 with sampling rates below 1.000 Hz, and three for overall bad data quality. Furthermore, two patients with primary generalized epilepsy syndromes and 13 with inconclusive diagnoses were excluded. The final sample comprised 24 participants aged between 19 and 62 years (*M* = 32.13, *SD* = 13.42). Detailed information on the patient sample is provided in Table 1.

**Table 1:** Patient information.

**ID**	**Sex**	**Age**	**E?**	**Seizure type**	**Seizure symptoms**	**AoO**	**Interictal EEG (EMU)**	**#sz**.	**MRI**	**OP**	**Focus**	**EF**	**#sHFO**	**COR**	**#tHFO**	**Keys**
P18	M	51	Yes	FIA, FBTC	n/a	43	Interm. theta T-R ant., sh-w. T-R (N3)	10	HyS Amy-R	No	T-R	R	0	–	–	–
P21	M	54	Yes	FA, FIA	Motor, epig., psy.	19	T slowing, sh-w. + spk. T-R	1	Negative	No	T-R	R	17	Yes	3/8	260
P22	M	29	Yes	FBTC	dysm.	28	sh-w. T-L posterior	4	Negative	No	F-L	L	8	Yes	–	–
P23	F	25	Yes	FIA, FBTC	psy., sens., veg., visual	7	sh-w, + spk. F	2	HIP asym. L < R	No	T-L	L	7	Yes	–	–
P25	M	21	No	NP, syncope	Motor	20	Interm. slowing T-R	0	HIP asym. R < L	No	–	N	2	–	13/1	801
P26	M	19	Yes	FA, FBTC	Motor, sens.	13	Poly-spk.-wave parox. F (R)	44	Negative	No	T-R	R	8	Yes	6/4	509
P27	F	30	Yes	FA, FIA, FBTC	Motor, dysm., epig.	22	sh-w. F-T	1	heter. O (NF1), PMG	No	TC-B	B	23	Yes	–	–
P29	M	24	Yes	FIA, FBTC	Motor, epig.	21	spk. T, interm. Sh-w. T-R	5	T-L un.	No	T-B	B	7	No	–	–
P32	M	21	Yes	FIA, FBTC	epig., vertiginous	19	sh-w. T-L	7	Negative	No	T-L	L	14	No	–	–
P33	F	35	Yes	FA, FBTC	Vertiginous	24	None	0	Negative	No	L	L	5	No	–	–
P37	F	25	Yes	FA, FBTC	psy., sens.	2	Interm. theta F-C	0	FCD HIP-L, F-L un.	No	T-L	U	20	(Yes)	–	–
P38	M	22	Yes	FA	Motor	20	sh-w. F	5	gangl. (1) HIP-R	Yes	F-R	R	4	Yes	3/12	229
P41	M	35	Yes	FA, FBTC	epig.	35	delta-theta with sh-w. T-L	2	heter.O (NF1)PMG	No	T-L	L	10	No	–	–
P42	F	19	Yes	FIA	n/a	19	asym. s-spind (L>R) + v-wav. (R>L)*	5	Pineal gland cyst	No	FC,	U	2	(Yes)	–	–
P43	F	62	Yes	FIA, FBTC	Motor, veg.	55	Parox. alpha with spk. + sh-w. FC	0	Negative	No	F-B	B	23	Yes	–	–
P44	F	46	No	NE, migraine	Dysm., vertiginous	n/a	Interm. slowing ant. L	0	Negative	No	•T-L	N	9	(No)	–	–
P45	F	29	Yes	FA, FIA, FBTC	n/a	11	Interm. theta + spk T posterior	0	Pineal gland cyst	No	TPO-L	L	5	Yes	–	–
P46	M	54	No	NE, syncope	Convulsive syncope	n/a	None	0	Negative	No	•FC-L	N	6	(No)	–	–
P47	F	21	No	NE, RMD	Motor	21	Interm. theta P-L	0	Negative	No	•TP-L	N	0	–	3/1	823
P52	F	23	No	Seizure free	n/a	18	None	0	DNET pOP: negative	Yes	°T-R	N	1	(No)	–	–
P53	M	52	Yes	FIA, FBTC	n/a	13	Interm. spk. F	6	heter. FTP-L	No	F-B	B	3	Yes	–	–
P59	M	24	Yes	FIA	n/a	22	Interm. slowing with sh-w. FT-R	3	FCD/tumor T-R	No	T-R	R	8	Yes	–	–
P61	M	31	No	NE, paroxysm	Motor	n/a	Interm. slowing FC-L	0	Negative	No	-	N	0	-	4/8	431
P62	M	19	Yes	FA, FIA, FBTC	psy., visual	3	sh-w.T-R posterior, polyspk TR (N3)	1	pOP: gangl. (1) T-R	Yes	PO-R	R	2	No	–	–

M, male; f, female; E?, epilepsy diagnosis; FA, focal aware; FIA, focal impaired awareness; FBTC, focal to bilateral tonic–clonic; NE/NP, non-epileptic/non-pathological; RMD, recurrent movement disorder; n/a, not assessable/applicable; epig., epigastric aura; psy., psychic; dysm., dysmnesic; sens., somatosensory symptoms/auras; veg., vegetative; AoB, age of onset; interm., intermittent; T, temporal; R, right; ant., anterior; mes., mesial; sh-w., sharp waves; spk, spikes; F, frontal; parox, paroxysm(ic); C, central; ^*^, asymmetric sleep spindles and vertex waves; P, parietal; #sz, number of epileptic seizures during stay; HyS, hyperintense signal; Amy, amygdala; HIP, hippocampal; heter., heterotopies, O, occipital; NF1, neurofibromatosis type I, PMG, polymicrogyria; FCD, focal cortical dysplasia; un., unclear; gangl., ganglioglioma (WHO grade 1); DNET, dysembryoplastic neuroepithelial tumor; pOP, post-operative; B, bilateral; focus, pathological focus; •, local dysfunction; °, seizure free after surgery; EF, epileptic focus group for statistical analysis (N, control; U, unclear); #sHFO, number of spontaneous HFOs during sleep; COR, HFOs correctly lateralized with the EF (in parentheses: correctly lateralized with focus if unclear or control); #tHFO, number of task-related HFOs (typing/resting) detected over the ROI-M. Keys, number of key presses during typing intervals.

After the HFO detection procedure, patients were assigned to the respective groups taking into account their diagnosis and the localization of the epileptic focus. For the purpose of this study, we defined the epileptic focus as the diagnosis made based on the seizure onset zone and/or the irritative zone (18). The epilepsy group contained 18 patients with focal epilepsy (seven women). The control group (*n* = 6, 3 women) consisted of five patients who underwent epilepsy monitoring but were finally diagnosed as not suffering from epilepsy, and one patient with post-surgical seizure freedom for 4 years without antiepileptic medication.

For the task-related HFO analysis, we systematically selected EEG segments with sufficient signal quality from a more homogeneous subsample, exclusively containing patients with motor symptoms to control for confounders. The final subsample included three epilepsy patients (three men, three right-handed) and three patients without epilepsy (two men, two right-handed).

### Data Acquisition

#### EEG Examination

A standard video EEG was recorded within the clinical procedure of epilepsy monitoring using Micromed System S.p.A. (Mogliano: Italy) with SystemPlus Evolution and an SD LTM 64 Express Amplifier (noise <0.15 V r.m.s CMRR >125 dB; input impedance >1,000 MΩ). Twenty-nine electrodes were placed according to the international 10–20 system (ground: Fpz; reference: Oz). Impedances were kept below 10 kΩ. The signal was high-pass filtered at 0.15 Hz (40 dB/decade anti-aliasing digital filters) and a 50-Hz notch was applied to cut out voltage noise. Data were digitized at a sampling rate of 1,024 Hz sampling rate, and in addition to the EEG channels, a differential electrocardiogram as well as electromyogram and electrooculogram were recorded.

#### Motor Task

During video EEG, cognitive testing was conducted at bedside in the mornings and evenings for overall six sessions including three tasks on verbal, spatial, and motor skill memory. The tasks were presented on a 17-inch screen using Neurobehavioral Systems Presentation (Version 18.1). Participants responded via keyboard.

For the task-dependent analysis at hand, we considered the two-part Finger Tapping task (19), which was used in six parallel versions, each comprising 12 learning and five recall trials of 30-s duration each, separated by intertrial resting of equal duration. A five-digit sequence of numbers associated with the four fingers—index to pinky—was displayed. Participants were instructed to repeatedly type the number sequence as fast and accurately as possible with their non-dominant hand and relax their fingers on the keys during resting. Each keypress and regular intervals during resting were marked by corresponding triggers in the EEG.

### HFO Identification

#### Data Selection

We targeted conditions of maximum HFO incidence and minimum noise. Early non-rapid eye movement sleep episodes with high-amplitude slow waves seem to facilitate the detection of pathological events (20). However, spontaneous HFO incidence can fluctuate around seizure occurrence (21) depending on seizure onset types (22) and is reduced by medication (23, 24). We extracted 20-min EEG segments—ideally providing early slow wave sleep episodes, maximal time distance to seizures, from the night of maximal drug taper. Furthermore, we extracted 12-min segments of highest data quality recorded during task performance. The trigger record was blinded before HFO identification. For semi-automated detection, we used MEEGIPS (25). Channels were referenced in a bipolar montage, as previous studies reported beneficial effects in terms of a reduced degree of non-cerebral noise related to ocular and facial artifacts (26–28).

#### Semi-Automated HFO Detection

We considered events that exhibit (a) a minimum frequency of 80 Hz, (b) at least four consecutive oscillations of regular morphology in the filtered signal, and (c) amplitudes distinguishable from the background signal (3, 4). We adhered to the typical classification by HFO type (1).

In a first step, automated detection was performed. A finite impulse response filter was applied with an 80–500 Hz band-pass. A root mean square (RMS) detector identified events of interest (EoIs) proportionally to the RMS mean background signal (29). Events that exceeded (i) an onset and offset transition threshold of 1.4 SD and (ii) a peak threshold of 3 SD above the RMS amplitude, and exhibited (iii) a minimum duration of 12 ms, were adopted. Consecutive EoIs separated by ≤ 30 ms were merged into one event. A Stockwell-transform power spectral density classifier was applied to filter EoIs for possible artifacts and to classify the residual events by HFO type (30); we adopted the original settings. EoIs were labeled according to their peak frequency as ripple (<250 Hz) or fast ripple (>250 Hz).

In a second step, we vertically split the screen for review (3, 4)—left, the filtered EEG at an expanded scale (450 mm/s, 10 μV, 3 s/view); right, the raw EEG (60 mm/s, 50 μV, 1 s/view). EoIs that (i) exhibited at least four consecutive oscillations of regular morphology in the filtered EEG and its Empirical Mode Decomposition and (ii) revealed an isolated blob in the Continuous Wavelet Transform distinguishing HFOs from false ripples (31) were considered HFOs. Per definition, HFOs must also stand out from the background. We considered this criterion fulfilled by the RMS detector algorithm as poor contrast of amplitude levels can be expected in scalp EEG (12). EoIs associated with artifacts were discarded. After a double check, temporal, local, and spectral HFO characteristics were exported for further analysis.

### Statistical Analysis

**Figure 1:**
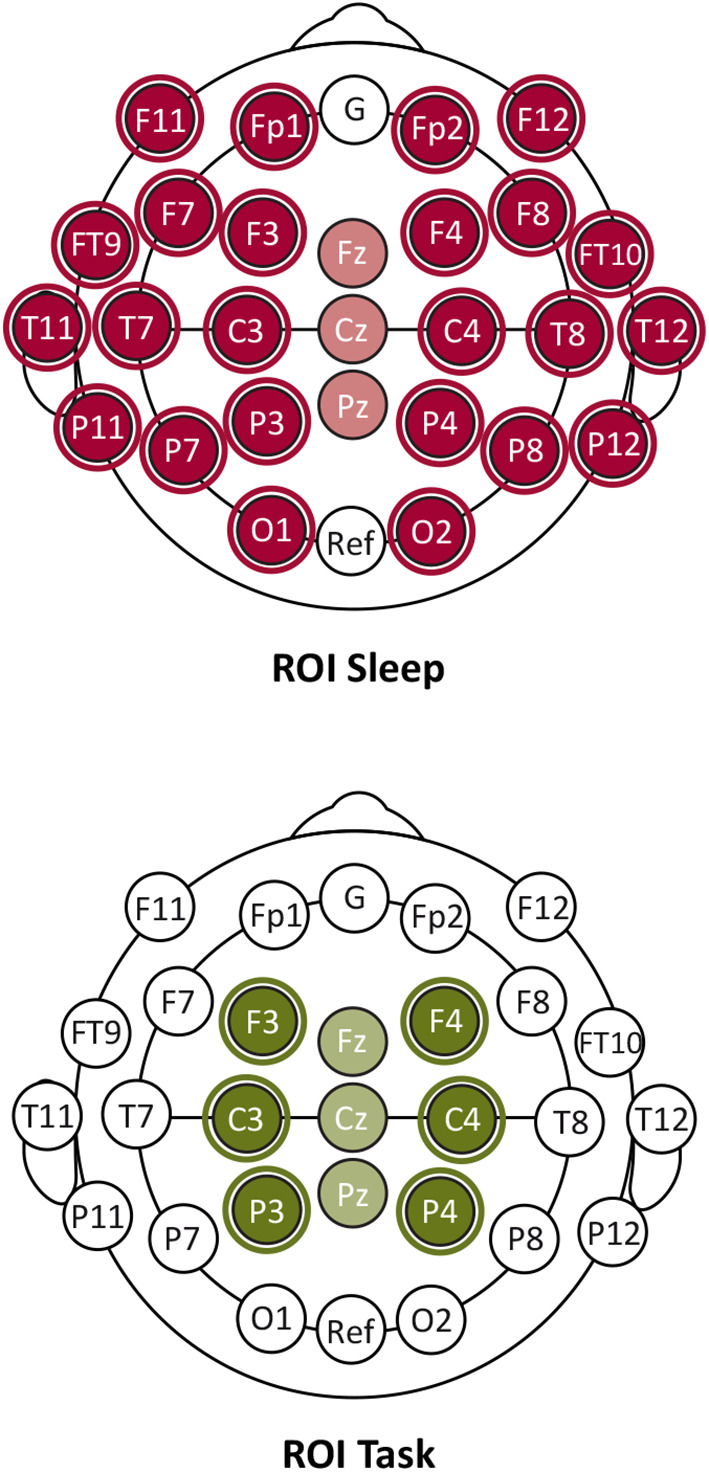
Regions of interest (ROI) electrode maps for the analysis of HFO occurrence during sleep (ROI Sleep) and motor task performance (ROI Task).

Statistical analyses were carried out using R (Version 3.4.2) and MATLAB R2017b. Semi-parametric comparative analyses on mean HFO rates were conducted using the R package MANOVA.RM (Version 0.2.39) (32). Results are reported referring to Wald-type statistic (*WTS*) including the parametric bootstrap *p*-value, and the degrees of freedom of the central χ^2^ distribution. Due to the small sample size, we indicate the resampled *p*-values as recommended by the authors (32). The significance level (α = 0.05) was adjusted for multiple comparison using Bonferroni–Holm correction. Spontaneous and task-related HFO occurrence rates were analyzed separately for the respective regions of interest (ROI, see Figure 1).

#### Analysis 1: Spontaneous HFOs During Sleep

Mean HFO rates per minute were calculated for each patient (a) across the total number of ROI Sleep channels and (b) across the number of channels grouped by cerebral hemispheres excluding midline channels. Spontaneous HFO incidence was analyzed with respect to epilepsy diagnosis (Epilepsy) and regarding a topographical correspondence with the epileptic focus laterality (Epileptic Focus). Cases with an undefined epileptic focus were excluded from this analysis.

#### Analysis 2: HFOs During Motor Task Performance

Intervals of 400 samples around each typing and resting trigger were scanned for HFO onsets within the corresponding ROI Task defined by central and adjacent electrodes. Events coupled with triggers were labeled accordingly as “typing HFO” or “resting HFO.” Unrelated events were discarded. Trigger-normalized HFO rates were calculated for each patient across all trials and intertrial resting periods by dividing the sums of (i) typing HFOs and (ii) resting HFOs by the respective trigger number. Task-related HFO occurrence was analyzed considering ROI Task hemispheres and the moving hand laterality. Midline channels were excluded.

## Results

### Analysis 1: Spontaneous HFOs During Sleep

Spontaneous HFOs were detected across the overall ROI Sleep with an average rate of 0.017 events/min (*SD* = 0.016, *N* = 24) and found to be increased in patients with epilepsy (*M* = 0.021, *SD* = 0.016, *n* = 18) compared to the control group (*M* = 0.007, *SD* = 0.008, *n* = 6).

#### Spontaneous HFOs and Epilepsy Diagnosis

**Figure 2:**
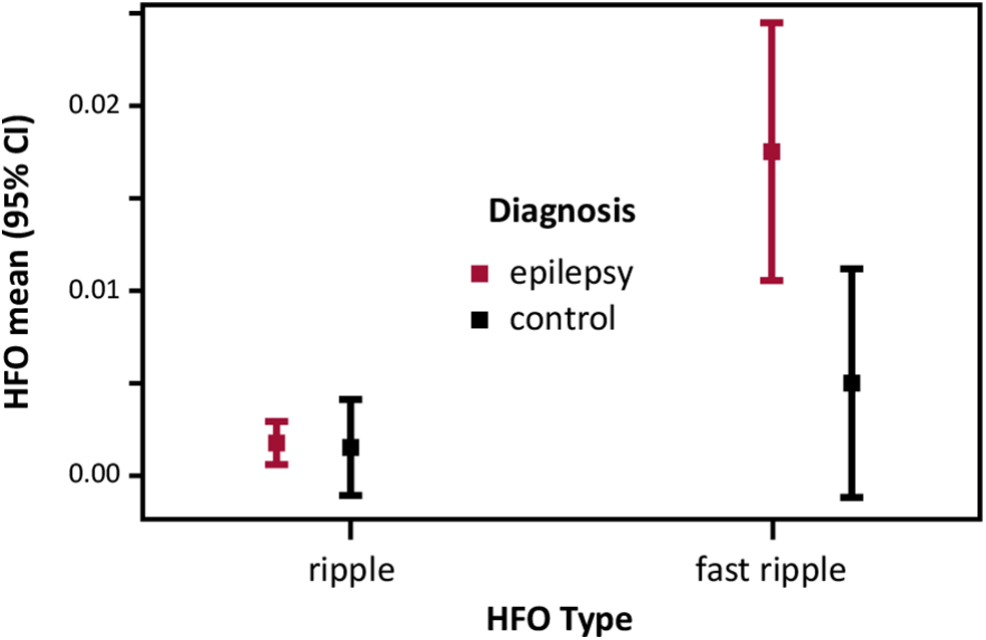
Spontaneous HFO incidence in patients with epilepsy and controls: HFO mean (95% CI) rates per minute recorded during sleep over ROI Sleep.

We examined the main effects and the interaction effect of the between-subject factor Epilepsy (epilepsy vs. control) and the within-subject factor HFO Type (ripple vs. fast ripple) on mean HFO rates. Results showed a statistically significant interaction between Epilepsy and HFO Type on mean HFO rates (WTS = 9.594, df = 1, *p* = 0.00538, *N* = 24). There was a statistically significant main effect of HFO Type (WTS = 23.370, df = 1, *p* = 0.00009, *N* = 24). The main effect of the factor Epilepsy (WTS = 7.411, df = 1, *p* = 0.0137) did not meet the level of significance after Bonferroni–Holm correction for multiple testing. Figure 2 illustrates increased HFO means in the fast ripple band compared to the ripple band in the epilepsy group compared to the control group. The CIs for all factor level combinations overlapped.

#### Spontaneous HFOs and the Epileptic Focus

**Figure 3:**
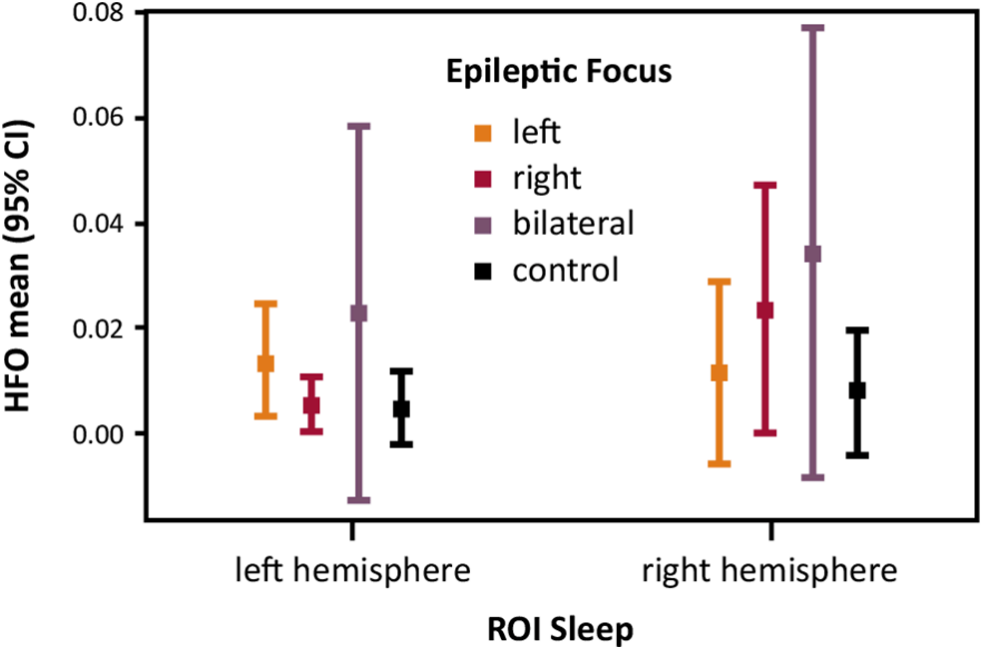
Spontaneous HFO incidence and the epileptic focus laterality: HFO mean (95% CI) rates per minute recorded during sleep over ROI Sleep hemispheres.

We examined the main effects and the interaction effect of the four-level between-subject factor Epileptic Focus (left vs. right vs. bilateral vs. control) and the two-level within-subject factor ROI Sleep (left vs. right) on mean HFO rates. Results showed no statistically significant interactions and main effects. Descriptive statistics indicate trends of HFO means topographically concordant with the epileptic focus in two out of three patients of the epilepsy subgroup as well as with the absent epileptic focus in the control group (see Figure 3). The CIs of all factor level combinations overlapped.

### Analysis 2: HFOs During Motor Task Performance

Task-related HFOs were detected over ROI Task with an average rate of 0.16 events/min (*SD* = 0.09, *N* = 6): with 0.20 events/min in the epilepsy group (*SD* = 0.10, *n* = 3) and 0.12 events/min in the control group (*SD* = 0.08, *n* = 3). Of the total HFO events detected, 72.81% were coupled either with typing triggers (41.86%) or resting triggers (30.95%). Trigger-unrelated events were excluded from further analysis.

**Figure 4:**
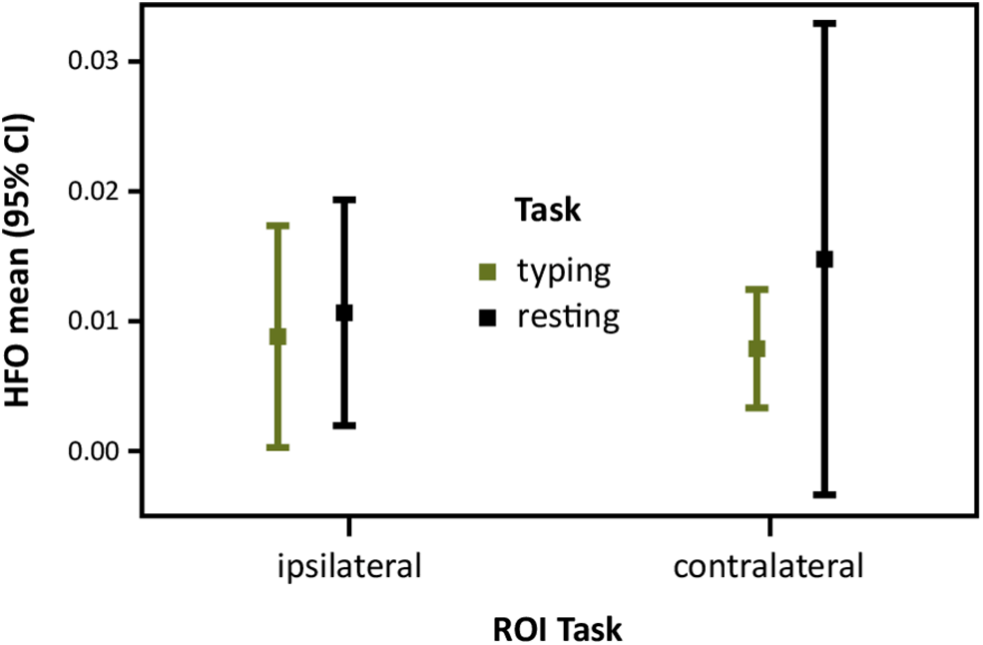
Task-related HFO occurrence in patients with motor symptoms: Trigger normalized HFO mean (95% CI) rates over ROI Task hemispheres ipsilateral and contralateral to the moving hand during typing trials and intertrial resting.

We examined the main effects and the interaction effect of the two-level within-subject factors Task (typing vs. resting) and ROI Task hemispheres (ipsilateral vs. contralateral) on mean HFO rates. Results showed no statistically significant interactions and main effects. Descriptive statistics indicate a trend of increased HFO means during resting (see Figure 4).

Total numbers of HFO events and comprehensive results including the HFO distributions in single cases can be found in the Supplementary Material (Tables S1–S9 and Figure S1).

## Discussion

The study at hand aimed at determining whether spontaneously occurring HFOs can be detected in scalp EEGs. In order to address this question, two hypotheses had been formulated: (i) it is possible to identify spontaneously occurring HFOs over brain areas that correspond with the epileptic focus in patients with epilepsy, and (ii) HFOs induced by self-paced motor activity in movement-associated central brain areas can be observed independently of an epilepsy diagnosis.

Both hypotheses relating the clinical and functional significance of HFOs to corresponding topographical activation patterns were not supported by our data: Firstly, spontaneous HFOs were not consistent with the epileptic focus. However, in line with previous findings (33, 34), we found spectral differences in scalp HFO distribution between epilepsy patients and controls, suggesting that prominent fast ripple rates indicate epileptic activity. Secondly, unlike previous findings (16, 17), task-related HFOs were not consistent with the movement-associated brain area. We found elevated HFO rates during periods of resting between the trials but not during finger tapping, notably in epilepsy patients compared to controls.

### Possible Biases

Considering the challenges of scalp HFO detection (10–12), any statistically significant result must be interpreted cautiously. Variations in general HFO incidence might correlate with signal quality rather than pathology. Such variations are stressed by the method of supervised detection: an increased false positive detection ideally leads to increased EoI rejection during visual review. Our data mirror this phenomenon, even showing a reversal of HFO rate differences between patients with epilepsy and controls when comparing the results of the automatic detection prior to and after visual review (see Table S10).

Another important bias stems from differences between single subjects. Even homogeneous samples have been shown to not lead to reliable HFO detections on the scalp (4, 35, 36), and also the presence of an epilepsy syndrome cannot be considered a valid predictor of successful scalp HFO detection (37). Varying signal-to-noise ratios between recordings introduce considerable errors into scalp EEG investigations. This effect only increases when considering automatic HFO detection in long-term recordings, given that the signal-to-noise ratio may differ not only between subjects but also between different recording days within the respective subjects.

Automatic detection increases these by magnifying the rates of false-positive detections deriving from low signal-to-noise ratios. Our experiences reflect these issues. Despite all attempts to adapt the detector configurations, EoIs were mainly not outstanding from the background, which has been previously attributed to false positives (38). Considering the outcome when using different configurations of the automatic detector, the detector appears to be highly sensitive to signal quality. We provide examples of this effect in the Supplementary Material (see Figure S2). After considering several adjustment settings, we did choose a rather sensitive approach in order to receive a large number of EoIs for visual inspection. However, the visual review may not compensate for this, leading to low specificity. The implementation of special measures during EEG acquisition and data preprocessing may be an essential prerequisite (39–43).

Furthermore, our findings regarding the ripple-to-fast ripple ratio must be regarded critically. Fast ripples seem to be a better indicator of epileptogenicity than ripples (1, 33). Nonetheless, our findings can be also explained by unreliable HFO identification. Even though parallel subdermal recordings underpin the detectability of fast ripples on the scalp, their incidence is very low (36). Surprisingly, fast ripples were more frequently detected than ripples across our sample (seeTable S4), indicating another possible bias. To put it into perspective: By virtue of their dynamics, fast ripples might appear superimposed on ripples (13). EoIs of one HFO type may remain either undetected or significantly underrepresented when using common RMS detector settings for both HFO types.

Additionally, there is evidence that EoI peak frequency calculations are reliant upon EEG acquisition settings such as sampling rate (44). As has been shown, filtering the EEG signal recorded at relatively low sampling rates can lead to peak frequencies being shifted from the ripple to the fast ripple band and vice versa (44). While it is common in standard EEG assessments, especially during long-term video EEG monitoring to use a similar sampling rate, a higher sampling frequency might be immanent when wanting to use the obtained data for HFO analyses. In addition, spatial sampling has been shown to be a key factor for the successful detection of HFOs (4). Similar to the sampling rate, also spatial sampling in this study did not meet standard suggestions from the community for an accurate detection of HFOs. This constitutes another bias to our findings and does further outline the special requirements for HFO detection, often unmet in standard clinical EEG assessments.

### Mediating Factors for HFO Occurrence

Yet, presuming the cerebral origin of all identified events, various factors may have further influenced the outcome of spontaneous HFO analysis: The heterogeneity likely influenced our inconsistent findings, as can be seen by the more conclusive trends observed in the relatively homogeneous subsample chosen for the motor task analysis (see Figure S1). It is important to note that one participant (P52), who does not meet the current criteria for epilepsy being resolved (45), was among the members of the control group with the lowest HFO rates; hence, it cannot cause a bias to our statistics, but rather exemplifies surgical success and the indicative potential of absent or sparse HFO incidence.

It has been shown that spontaneous HFO incidence varies across different types of epilepsies and seizures (23), and with seizure frequency (9). HFOs may be a less specific but more ambivalent marker than hitherto assumed: Events were associated with the seizure onset zone (3), the irritative zone (6, 46, 47), the underlying lesion (48, 49), and, in the context of motor seizure symptoms, the Rolandic areas (8). Alternatively, varyingly deep located sources explain sparse HFO identification on the surface (48).

There are indications that HFOs are a dynamic rather than a constant measure, closely linked to seizure occurrence (21, 23, 50, 51). Due to different findings, there is no clear evidence on their relationship with seizure frequency though (9, 52). In our sample, the number of general seizure records was higher in the epilepsy group compared to the control group, notably in patient P26 who exhibited a prominent focus-concordant HFO pattern. Furthermore, fluctuations in HFO incidence may also be related to seizure symptoms (8).

Another mediating factor could be the antiepileptic medication. We chose EEG segments from later sessions, when antiepileptic drug taper was maximal in the EMU. However, little is known yet about the effects of different drugs on HFO occurrence. Changes in HFO occurrence have been reported after the administration of different drug types (23, 24, 53). Reduced HFO amplitudes, in particular, were related with pharmacologically induced GABAergic and cholinergic changes (54, 55).

Finally, age may also construe a confounding factor (13), as previous studies suggest a U-shaped developmental HFO occurrence across the life span with the lowest rates in young adults between 19 and 32 years (56, 57). Similar age-related trends can be observed in our sample (Table 2).

**Table 2:** Age-related differences in HFO occurrence.

**Condition**	**Age**	***n***	**HFO means**	**SD**
Sleep	≤ 32 years	16	6.94	6.83
	>32 years	8	9.13	7.59
Task	≤ 32 years	5	105.00	45.43
	>32 years	1	189.00	–

### Movement-Related Scalp HFOs

To our knowledge, this is the first study that analyzed movement-related scalp HFOs up to the fast ripple range in patients with and without epilepsy. Previous findings in the ripple range suggest a common contralateral activation pattern in epilepsy patients and healthy adults (16, 17). We found inconsistent HFO patterns with a trend toward higher HFO occurrence during rest compared to typing.

Two explanations support a reduced HFO detection during the active phases: First, it is likely that with decreasing signal-to-noise ratio, the small-scale events become indiscernible in the EEG. Findings of consistent absolute amplitude levels when comparing evoked events recorded at the edges of identification threshold (58) support this assumption. On that note, also the design of the task consisting of blocks of self-paced repetitive finger movements may have fostered this effect, as movement-related high-frequency evoked activity has been reported to be strictly time-locked to single finger movements and rather brief in duration (59). Second, amplitude diminution during stimulation has been associated with interference effects (60, 61) and homeostatic plasticity (40, 62) in both patients and healthy subjects.

Furthermore, it is advisable to take into account possible additional influences when it comes to interpretation of the motor task results. The task was rather complex and HFO fluctuations may be linked to factors such as typing speed (63, 64), or previous motor skill acquisition and working memory capacity (65, 66). During intertrial rest, which included a countdown and a starting cue, HFO occurrence may also be related to attention and motor preparation (67, 68). In our sample the typing score did vary across patients: On average, the control group scored higher (*M* = 685, *SD* = 220.25, *n* = 3) than the epilepsy group (*M* = 332.67, *SD* = 153.49, *n* = 3), indicating increased typing speed.

Alternatively, cognitive as well as motor functioning may have been affected by pathologies in some patients, thus mediating task-related HFO incidence. Cognitive deficits affecting motor planning, coordination, attention, and response inhibition must be considered when using cognitive tasks. For instance, P38 with frontal lobe epilepsy exhibited a very low typing score and a lower task-related HFO rate. In addition, subjects P47 and P61 of the control group presented with motor symptoms and local dysfunction; in particular, P61 exhibited an extensive comorbidity. It cannot be excluded that HFOs relate to different pathologies (58, 69–73).

In the context of epilepsy, fMRI findings showed a notable co-activation of the motor system, positively correlated with non-motor task difficulty and myoclonic seizure frequency (74). Similarly, abnormal EEG activity was observed during motor planning associated with frequent focal motor seizures (75). We speculate that the cognitive effort required for task performance may have provoked disease activity, temporally overlapping with intertrial resting intervals. Moreover, with regard to HFO amplitude levels, interferences with such pathological activity may account for indiscernible HFOs during task performance in task-positive areas, whereas the effect may vanish off-task.

Finally, our sample showed inconsistently lateralized activations during task performance. There is one study that supports the notion of a generally asymmetric hemispheric HFO activation—contralateral of the moving hand—regardless of the side of movement (68). However, handedness and dexterity may have strong impact (76–80), particularly when performing a motor task with the non-dominant hand—as done in our study—transient reversal of hemispheric asymmetry may be provoked (81).

In summary, physiological and pathological HFO occurrence may overlap, cumulate, or interfere. Divergent HFO patterns during task performance may indicate an influence of disease activity, functional restrictions, or compensation (see Table S11). To date, there is no direct evidence on pathological alterations of HFO laterality during motor task performance. However, findings of TMS studies indicate reduced lateral inhibition in the motor cortex after stimulation that was associated with epileptic focus laterality, even when located at distance (82), and with bilaterally spreading myoclonic activity (83). Conclusions cannot be drawn based on our data; the laterality of the epileptic focus consistently overlapped with the task-associated hemisphere. In a clinical context, making use of a statistical parametric mapping approach may help to overcome the problem of overlapping physiological and pathological HFO populations by taking into account the normative mean of event occurrences (84).

### Limitations and Future Implications

**Figure 5:**
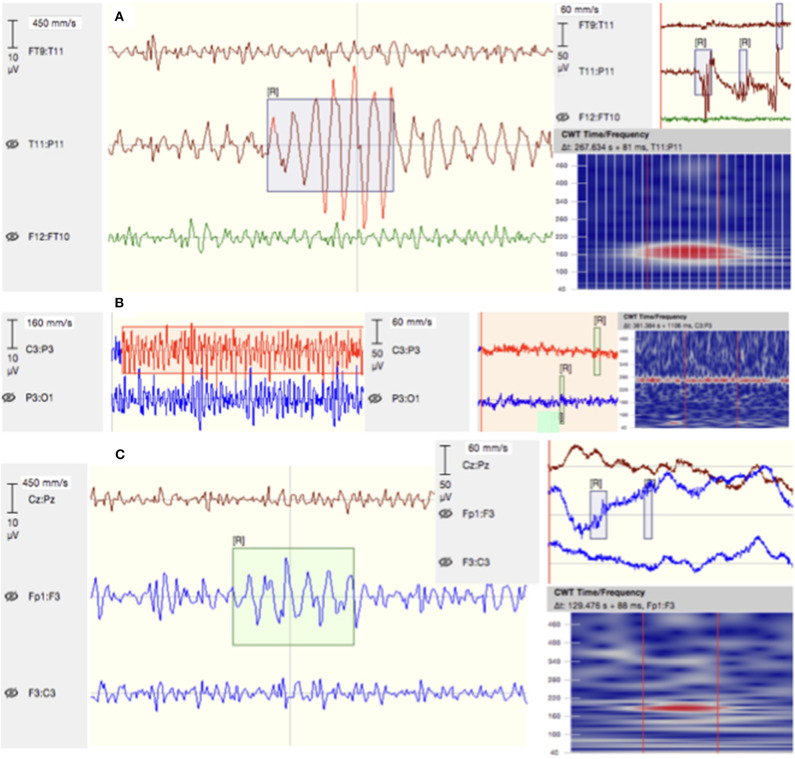
Examples of detected EoI. **(A)** A “false” ripple due to a filtered artifact detected over T11–P11 in patient P53. **(B)** Background noise mimicking high-frequency activity in the filtered EEG signal of patient P21. **(C)** A ripple embedded in noise on channels Fp1–F3 of patient P23. Given are signals filtered between 80 and 500 Hz (left), the raw signal (right), and their respective time–frequency plots.

The confusingly similar characteristics of HFOs and artifactual components pose a major challenge on the identification process (see Figure 5). In this context, line-voltage harmonics as well as their respective derivates mimic patterns that can easily be confused with the suggested continuous physiological high-frequency activity (85). This underpins the necessity of a strict control for confounders.

Yet, the standard acquisition setting within clinical routine settings, such as the epilepsy monitoring unit, hardly provides ideal conditions for recording HFOs: spatial sampling (4), sampling rate (44), impedances, and signal amplification (41) must be reconsidered for future analyses, as they might need to be optimized when attempting to detect HFOs. Special regard deserves artifact prevention at first hand, considering operable cognitive paradigms that meet clinical requirements.

Although semi-automated detection reduces the risk of false-positive detection (38), its reliability is limited. Good test–retest reliability was stated for fully automated HFO detection in intracranial EEG (86). However, most detectors were tested on intracranial recordings only, thus limiting their validity, when confronted with artifacts encountered in scalp recordings (87). Our EoI marker statistics of initial test runs indicate high noise sensitivity (see Figure S2). Individual threshold specification may be more efficient, although not compensating for variations in data quality, particularly in long-term recordings. Moreover, HFO detection in the broad band appears to be problematic, suggesting analyses of narrower frequency bands to improve reliability.

The visual review does not compensate for false negatives; and our single-rater approach neither improved reliability. Good interrater reliability would be desirable; however, ambiguous findings were reported (88, 89). Visual HFO identification lacks objectivity. A blinded approach is rarely reported, considering the visibility of abnormal activity in EEGs. We blinded the review for diagnoses and trigger record; a completely objective analysis may only be realized by fully automated detection though.

The validity of scalp HFO analysis for epilepsy screening is debatable: Due to our study design, we opted to investigate HFOs independently from traditional markers, which may have affected the detection specificity. The added clinical value of HFOs filtered by their temporal co-occurrence with traditional markers, as commonly done, is unclear; however, cross-rates may improve clinical validity (90).

## Conclusion

The present study demonstrated the technical and methodological obstacles scalp HFO analysis faces when it comes to application. Since noise is an essential confounder, scalp HFO analysis requires high-quality data acquired within a highly controlled setting. These prerequisites might not be easily met within the typical clinical routine. General drawbacks fundamentally cut the reliability, validity, and generalizability of findings; current HFO research basically requires transparency and standardization. Conclusions on the validity of HFO analysis cannot be drawn unless the operating procedure is reported in detail. A significant milestone toward successful clinical application is a reliable automatic detection for reasons of objectivity and economy. Since detection algorithms are reliant upon initial expert marking, international efforts toward standardization are required to answer the central question: Is there a signal within the noise?

## Data Availability Statement

The datasets generated for this study are available on request to the corresponding author.

## Ethics Statement

The studies involving human participants were reviewed and approved by Ethikkommission für das Bundesland Salzburg (local ethics committee of the county Salzburg). The patients/participants provided their written informed consent to participate in this study. Written informed consent was obtained from all patients for the publication of any indirectly identifiable data included in this article.

## Author Contributions

This study was planned by YH and ET. YH supervised the overall study conduct, as well as data analysis. NG analyzed the data, performed the statistical analysis, and prepared the first manuscript (parts were used as a basis for her Master's thesis). AM conducted the experimental tasks during the EEG recordings. AT supervised HFO detection and revised the final manuscript for publication. All authors read and agreed upon the final version prior to submission.

## Conflict of Interest

The authors declare that the research was conducted in the absence of any commercial or financial relationships that could be construed as a potential conflict of interest.
