# Supplementary material for: Pitfalls in Scalp High-Frequency Oscillation Detection From Long-Term EEG Monitoring
Source: Front Neurol. 2020 Jun 2;11:432. doi: 10.3389/fneur.2020.00432 (PMC7280487; doi:10.3389/fneur.2020.00432)
Supplement: Supplementary file 1 [file Table_1.DOCX]

Supplementary Material:

Pitfalls in scalp high-frequency oscillation detection from long-term EEG monitoring.

Nathalie Gerner^1,2^, Aljoscha Thomschewski^1,2*^, Adrian Marcu^1^, Eugen Trinka^1^, Yvonne Höller^1,3^

^1^Department of Neurology, Christian-Doppler Medical Centre and Centre for Cognitive Neuroscience, Paracelsus Medical University, Salzburg, Austria

^2^Department of Mathematics, Paris-Lodron University of Salzburg, Salzburg, Austria

^3^Department of Psychology, University of Akureyri, Akureyri, Iceland

*** Correspondence:**Aljoscha Thomschewski
[a.thomschewski@salk.at](mailto:a.thomschewski@salk.at)

# SUPPLEMENTARY TABLES

**Table S1**: Total number of spontaneous HFOs detected in sleep EEG segments.

|  | **Night recording 18:00-06:00** | | | **EEG segment** | |  | **HFO identification** | |
| --- | --- | --- | --- | --- | --- | --- | --- | --- |
| **ID** | Night | Seizure (n) |  | Start time | Duration (s) |  | EoIs (n) | HFO (n) |
| P18 | 1 | 0 |  | 23:25 | 1200 |  | 109 | 0 |
| P21 | 2 | 0 |  | 00:10 | 1200 |  | 970 | 17 |
| P22 | 2 | 0 |  | 02:26 | 1200 |  | 830 | 8 |
| P23 | 2 | 0 |  | 23:43 | 1200 |  | 861 | 7 |
| P25 | 2 | 0 |  | 04:41 | 1200 |  | 1132 | 2 |
| P26 | 3 | 9 |  | 03:07 | 1200 |  | 1100 | 8 |
| P27 | 2 | 1 |  | 03:29 | 1200 |  | 3964 | 23 |
| P29 | 2 | 1 |  | 22:18 | 1200 |  | 678 | 7 |
| P32 | 3 | 1 |  | 00:07 | 1200 |  | 2034 | 14 |
| P33 | 2 | 0 |  | 03:06 | 1200 |  | 2057 | 5 |
| P37 | 3 | 0 |  | 23:32 | 1200 |  | 1081 | 20 |
| P38 | 1 | 1 |  | 01:24 | 1200 |  | 860 | 4 |
| P41 | 3 | 2 |  | 22:49 | 1200 |  | 519 | 10 |
| P42 | 3 | 2 |  | 00:00 | 1200 |  | 518 | 2 |
| P43 | 3 | 0 |  | 04:51 | 1200 |  | 4139 | 23 |
| P44 | 1 | 0 |  | 00:21 | 1200 |  | 7613 | 9 |
| P45 | 2 | 0 |  | 00:31 | 1200 |  | 1001 | 5 |
| P46 | 3 | 0 |  | 03:47 | 1200 |  | 2712 | 6 |
| P47 | 3 | 0 |  | 01:12 | 1200 |  | 264 | 0 |
| P52 | 3 | 0 |  | 06:20 | 1200 |  | 901 | 1 |
| P53 | 2 | 2 |  | 23:12 | 1200 |  | 2694 | 3 |
| P59 | 2 | 1 |  | 00:22 | 1200 |  | 1676 | 8 |
| P61 | 3 | 0 |  | 06:16 | 1200 |  | 854 | 0 |
| P62 | 1 | 0 |  | 22:18 | 1200 |  | 980 | 2 |

*Sleep segments of 20-minute duration were selected according to the following priority: (1) Night of maximal anti-epileptic drug taper, (2) Availability of 20 minutes of sleep, preferably slow wave sleep from the earliest sleep cycle, (3) Best signal quality available, (4) Maximal interval between epileptic seizures.*

**Table S2**: Total number of HFOs detected in motor task EEG segments.

| **ID** | **Day** | **Session** | **Start time** | **Duration (s)** | **Handedness** | **EoI (n)** | **HFO (n)** |
| --- | --- | --- | --- | --- | --- | --- | --- |
| P21 | 3 | 5 | 17:37 | 726 | right | 1445 | 20 |
| P25 | 3 | 4 | 08:14 | 724 | right | 6504 | 17 |
| P26 | 3 | 5 | 18:24 | 725 | right | 713 | 11 |
| P38 | 3 | 4 | 08:12 | 728 | right | 3348 | 33 |
| P47 | 2 | 3 | 08:15 | 727 | right | 5572 | 4 |
| P61 | 2 | 3 | 18:30 | 759 | left | 1865 | 19 |

*Motor task segments of ~12-minute duration were selected according to the following priorities: (1) Learning session. (2) Best data quality available. (3) Trigger recordings available. Exclusion of P43, P44, P46, and P52 due to low data quality. HFO(n)* *= total number of HFOs detected over ROI-M prior to trigger analysis.*

**Table S3**: Two-way RM analysis - Spontaneous HFO incidence and epilepsy diagnosis.

| **Effect** | **Test statistic (WTS)** | **df** | **p-value (paramBS)** |  |
| --- | --- | --- | --- | --- |
| Epilepsy | 7.411 | 1 | .01370 |  |
| HFO Type | 23.370 | 1 | .00009 | ***** |
| Epilepsy × HFO Type | 9.594 | 1 | .00538 | * |

* significant after Bonferroni-Holm correction with *p* < 0.05.

**Table S4**: Descriptive statistics - Spontaneous HFO incidence and epilepsy diagnosis.

| **Group** | **n** | **Ripple Type** | **HFO means** | **lower 95% CI** | **upper 95% CI** |  |
| --- | --- | --- | --- | --- | --- | --- |
| epilepsy | 18 | ripple | 0.00162 | 0.00017 | 0.00307 |  |
|  |  | fast ripple | 0.01748 | 0.00938 | 0.02558 |  |
| controls | 6 | ripple | 0.00139 | -0.00365 | 0.00643 |  |
|  |  | fast ripple | 0.00486 | -0.00767 | 0.01739 |  |

*n = number of patients, CI = confidence interval.*

**Table S5**: Two-way RM analysis - Spontaneous HFO incidence and the epileptic focus.

| **Effect** | **Test statistic (WTS)** | **df** | **p-value (paramBS)** |  |
| --- | --- | --- | --- | --- |
| Epileptic Focus | 2.523 | 3 | .35203 |  |
| ROI Sleep | 1.923 | 1 | .14678 |  |
| Epileptic Focus × ROI Sleep | 3.507 | 3 | .42600 |  |

*ROI Sleep channels grouped by brain hemispheres; midline channels excluded.*

**Table S6**: Descriptive statistics - Spontaneous HFO incidence and the epileptic focus.

| **Epileptic Focus** | **n** | **ROI Sleep** | **HFO means** | **lower 95% CI** | **upper 95% CI** |  |
| --- | --- | --- | --- | --- | --- | --- |
| left | 6 | left | 0.013636 | -0.006939 | 0.034212 |  |
|  |  | right | 0.011364 | -0.021972 | 0.044700 |  |
| right | 6 | left | 0.005303 | -0.004861 | 0.015468 |  |
|  |  | right | 0.023485 | -0.022000 | 0.068970 |  |
| bilateral | 4 | left | 0.022727 | -0.060986 | 0.106440 |  |
|  |  | right | 0.034091 | -0.066640 | 0.134822 |  |
| control | 6 | left | 0.004545 | -0.008924 | 0.018015 |  |
|  |  | right | 0.007576 | -0.014874 | 0.030025 |  |

*n = number of patients, CI = confidence interval.*

**Table S7**: Two-way RM analysis of task-related HFO occurrence.

| **Effect** | **Test statistic (WTS)** | **df** | **p-value (paramBS)** |  |
| --- | --- | --- | --- | --- |
| Task | 0.890 | 1 | .36536 |  |
| ROI Task | 0.117 | 1 | .74004 |  |
| Task × ROI Task | 0.313 | 1 | .58493 |  |

*ROI Task channels grouped by brain hemisphere; midline channels excluded.*

**Table S8**: Descriptive statistics - Task-related HFO occurrence in the ROI Task.

| **Task** | **ROI Task** | **HFO means** | **lower 95% CI** | **upper 95% CI** |  |
| --- | --- | --- | --- | --- | --- |
| typing | ipsilateral | 0.007853 | -0.001112 | 0.016818 |  |
|  | contralateral | 0.008850 | -0.008422 | 0.026122 |  |
| resting | ipsilateral | 0.014740 | -0.021717 | 0.051197 |  |
|  | contralateral | 0.010609 | -0.006813 | 0.028030 |  |

*CI = confidence interval.*

**Table S9**: Topographical correspondence of spontaneous HFO incidence with the epileptic focus or non-epileptic pathological foci

| **ID** | **Group** | **Focus*** | **EF** | **#HFO** | **Distribution** | **Laterality** | **Location** |
| --- | --- | --- | --- | --- | --- | --- | --- |
| P18 | epilepsy | T-R | T-R | 0 | - | - | - |
| P21 | epilepsy | T-R | T-R | 17 | lateral | ✓ | 🗶 |
| P22 | epilepsy | F-L | F-L | 8 | lateral | ✓ | 🗶 |
| P23 | epilepsy | T-L | T-L | 7 | lateral | ✓ | 🗶 |
| P25 | control | - | - | 2 | midline | - | - |
| P26 | epilepsy | T-R | T-R | 8 | lateral | ✓ | 🗶 |
| P27 | epilepsy | TC-B | TC-B | 23 | multifocal | ✓ | 🗶 |
| P29 | epilepsy | T-B | T-B | 7 | frontocentral | 🗶 | 🗶 |
| P32 | epilepsy | T-L | T-L | 14 | bifocal | 🗶 | 🗶 |
| P33 | epilepsy | L | L | 5 | equal | 🗶 | - |
| P37 | epilepsy | TF-B | unclear | 20 | multifocal ^a^ | ✓^*^ | ✓^*^ |
| P38 | epilepsy | F-R | F-R | 4 | lateral | ✓ | 🗶 |
| P41 | epilepsy | T-L | T-L | 10 | bifocal | 🗶 | 🗶 |
| P42 | epilepsy | FC-B | FC | 2 | bifrontal | ✓^*^ | ✓ |
| P43 | epilepsy | F-B | F-B | 23 | multifocal ^a^ | ✓ | 🗶 |
| P44 | control | T-L | - | 9 | multifocal | 🗶^*^ | 🗶^*^ |
| P45 | epilepsy | TPO-L | T-L | 5 | lateral | ✓ | ✓^*^ |
| P46 | control | FC-L | - | 6 | lateral | 🗶^*^ | 🗶^*^ |
| P47 | control | TP-L | - | 0 | - | - | - |
| P52 | control^○^ | T-R^○^ | T-R^○^ | 1 | focal | 🗶^*^ | 🗶^*^ |
| P53 | epilepsy | F-B | F-B | 3 | bifocal ^a^ | ✓ | 🗶 |
| P59 | epilepsy | T-R | T-R | 8 | multifocal ^a b^ | ✓ | ✓ |
| P61 | control | - | - | 0 | - | - | - |
| P62 | epilepsy | PO-R | PO-R | 2 | bifocal | 🗶 | 🗶 |

*Spontaneous HFO incidence based on total HFO numbers. EF = epileptic focus; * pathological focus (epileptic and non-epileptic) and associated HFO incidence;* ***^○^*** *seizure free after epilepsy surgery;* ***^a^*** *dominant right-hemispheric HFO incidence;* ***^b^*** *dominant HFO incidence concordant with the epileptic focus.*

**Table S10**: Mean HFO occurrence in diagnostic groups during sleep (ROI Sleep) and motor task performance (ROI Task).

| **Condition** | **Group** | **n** | **M 1** | **SD 1** | **M 2** | **SD 2** |
| --- | --- | --- | --- | --- | --- | --- |
| Sleep | epilepsy | 18 | 1448.39 | 1137.07 | 9.22 | 7.22 |
|  | control | 6 | 2246.00 | 2754.55 | 3.00 | 3.69 |
| Task | epilepsy | 3 | 1835.33 | 1360.18 | 21.33 | 11.06 |
|  | control | 3 | 4647.00 | 2453.94 | 13.33 | 8.15 |

*Group HFO means (M) and standard deviation (SD) of n patients after automated detection (M1 and SD1) and visual review (M2 and SD2).*

**Table S11**: Individual differences in the motor symptom subsample.

| **EPILEPSY** | | |  | **CONTROL** | | |
| --- | --- | --- | --- | --- | --- | --- |
| **P21** | **P26** | **P38** |  | **P25** | **P47** | **P61** |
| Temporal lobe  epilepsy | Temporal lobe  epilepsy | Frontal  lobe  epilepsy | **Diagnosis** | POTS | Recurrent movement disorder | Asphyxia, hyper-metabolism |
| temporal right | temporal right  INS-SMA | frontal  right | **Focus** | - | ^●^ temporal-parietal left | ^○^ temporal medial bilateral |
| 2 | 64 | 16 | **Seizures (n)** | 1 | 0 | 2 |
| motor,  epigastric,  psychic | motor left,  sensoric | motor bilateral | **Symptoms** | motor | motor left | motor  bilateral |
| yes | yes | yes | **Ictal EEG** | no | N/A | no |
| no | INS right | HIP right | **Lesion** | no | no | no |
| 2 | 3 | 3 | **Medication** | 0 | 0 | 3 |
| N/A | N/A | N/A | **Comorbidity** | N/A | N/A | yes ^*^ |
| right | right | right | **Handedness** | right | right | left |
| 5 | 5 | 4 | **Task session** | 4 | 3 | 3 |
| 260 | 509 | 229 | **Typing score** | 801 | 823 | 431 |

*Seizures (n) = number of epileptic and non-epileptic seizures during the 4-day inpatient stay at the EMU. For diagnosis: POTS = postural orthostatic tachycardia syndrome. For Focus / Lesion: HIP = hippocampus, INS = insula, SMA = supplementary motor area, ^●^ local dysfunction, ^○^ hypermetabolism. For Comorbidity: ^*^ severe pneumonia, multiorgan dysfunction, cerebral edema.*

#
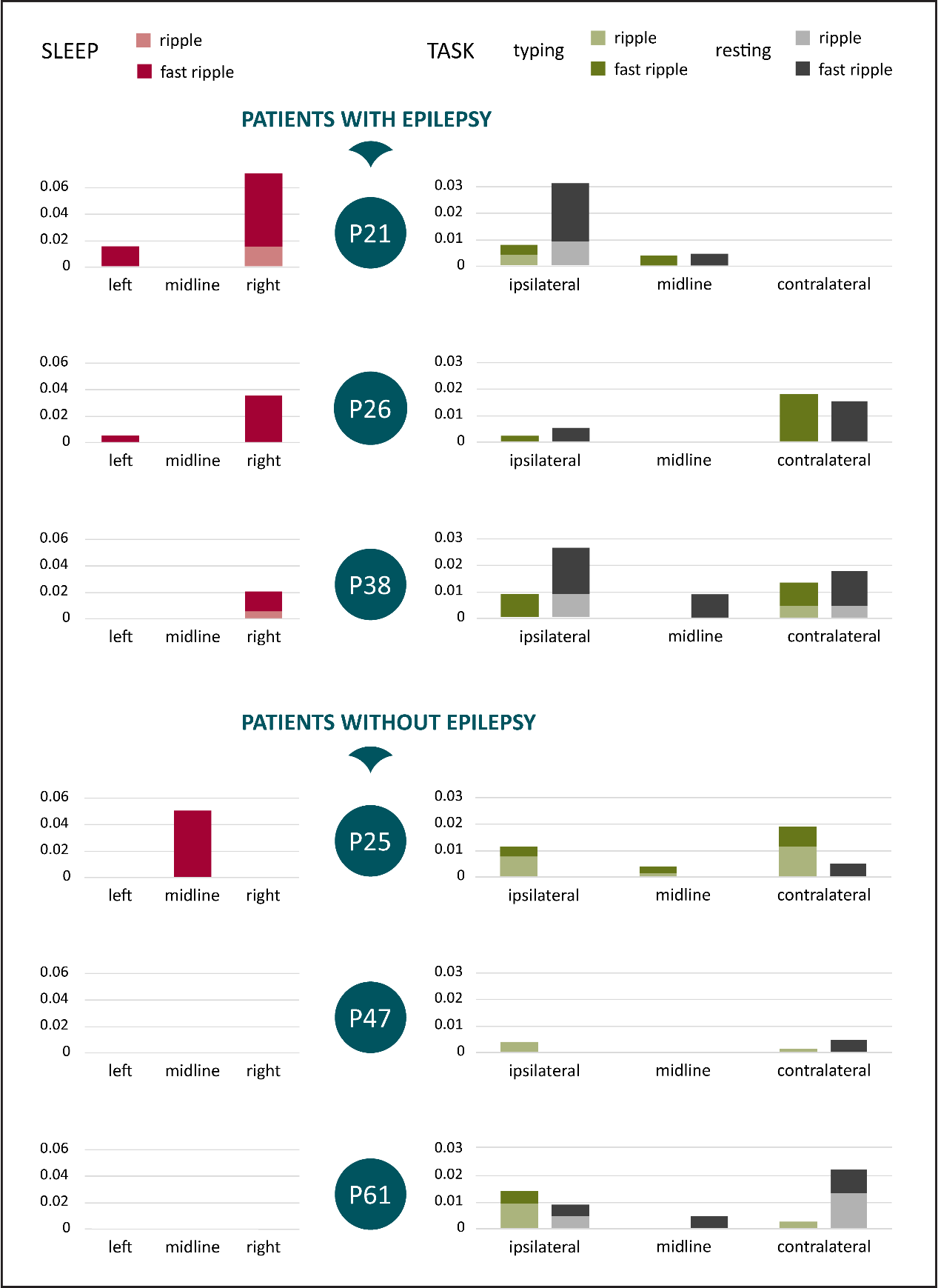
SUPPLEMENTARY FIGURES

**Figure S1**: HFO occurrence during sleep and motor task performance in single cases of the motor symptom subsample. Note that spontaneous HFO incidence refers to mean HFOs per minute, whereas task-related HFO occurrence refers to trigger-normalized HFO rates.

**
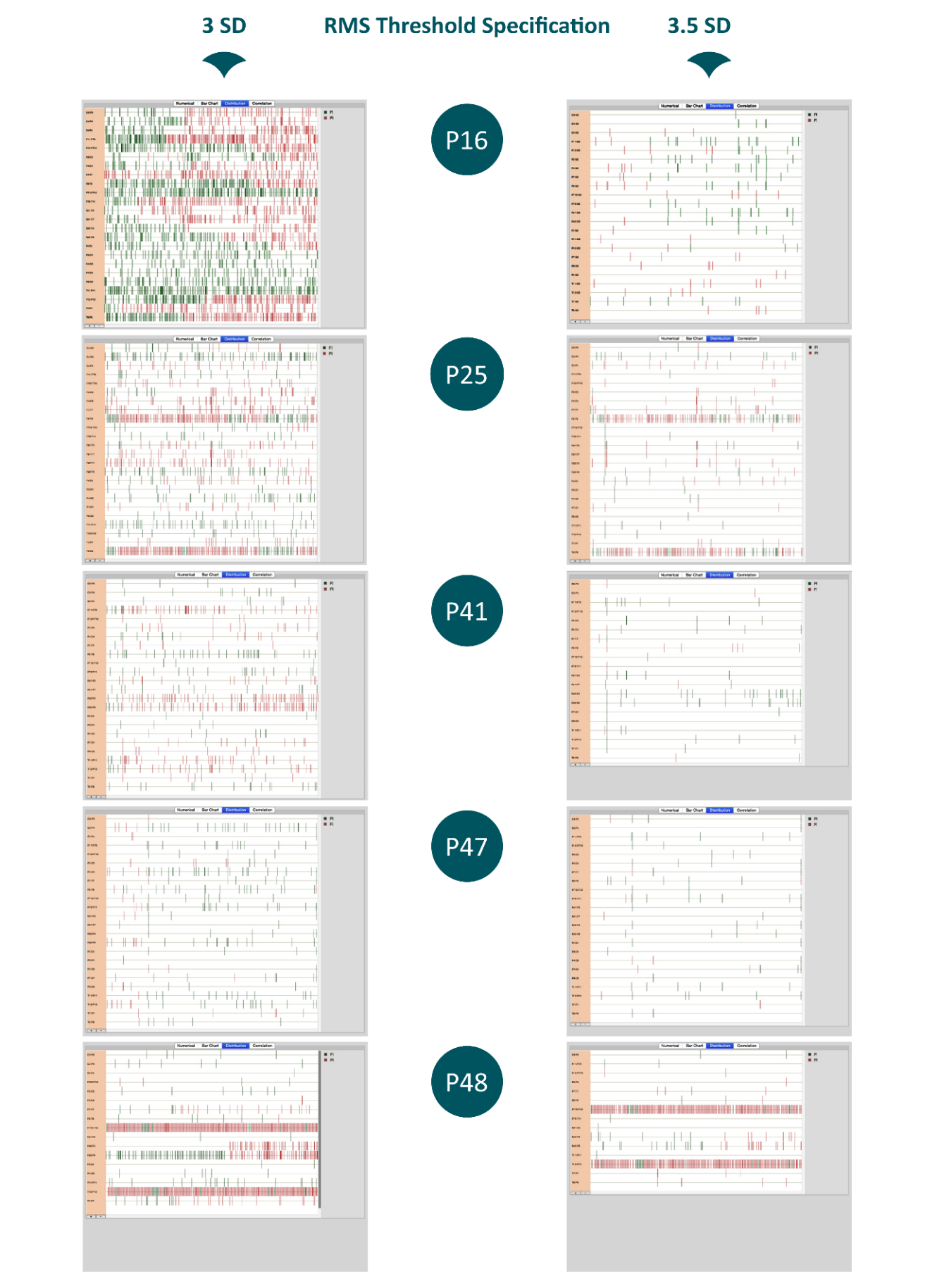
Figure S2**: Marker statistics show five examples of RMS amplitude threshold adjustment in 20-minute sleep segments. High EoI density and regular EoI distribution pattern reflect artefacts. SD = standard deviation.
